# Supplementary material for: Toward carbon neutrality: Uncovering constraints on critical minerals in the Chinese power system
Source: Fundam Res. 2022 Mar 3;2(3):367–74. doi: 10.1016/j.fmre.2022.02.006 (PMC11197575; doi:10.1016/j.fmre.2022.02.006)
Supplement: Supplementary file 1 [file mmc1.docx]

Toward carbon neutrality: uncovering constraints on critical minerals in the Chinese power system

Wendong Wei^a,b,c^, Zewen Ge^d^, Yong Geng^a,b,c^*, Mingkun Jiang^e^, Zhujun Chen^f^, Wei Wu^g^

^a^ School of International and Public Affairs, Shanghai Jiao Tong University, Shanghai 200030, China

^b^ SJTU-UNIDO Joint Institute of Inclusive and Sustainable Industrial Development, Shanghai Jiao Tong University, Shanghai 200030, China

^c^ China Institute for Urban Governance, Shanghai Jiao Tong University, Shanghai 200030, China

^d^ China-UK Low Carbon College, Shanghai Jiao Tong University, Shanghai, 201306, China

^e^ Key Laboratory of Pressure Systems and Safety (MOE), School of Mechanical and Power Engineering, East China University of Science and Technology, Shanghai 200237, China

^f^ Business School, University of Shanghai for Science and Technology, Shanghai 200093, China

^g^ Shanghai Climate Center, Shanghai 200030, China

*Corresponding author: [ygeng@sjtu.edu.cn](mailto:ygeng@sjtu.edu.cn) (Y. Geng).

**Abstract**

China has set up its ambitious carbon neutrality target, which mainly relies on significant energy-related carbon emissions reduction. As the largest important contributing sector, power sector must achieve energy transition, in which critical minerals will play an essential role. However, the potential supply and demand for these minerals are uncertain. This study aims to predict the cumulative demand for critical minerals in the power sector under different scenarios via dynamic material flow analysis (DMFA), including total demands, supplies and production capacities of different minerals. Then, these critical minerals are categorized into superior and scarce resources for further analysis so that more detailed results can be obtained. Results present that the total minerals supply will not meet the total minerals demand (74260 kt) in 2060. Serious resource shortages will occur for several key minerals, such as Cr, Cu, Mn, Ag, Te, Ga, and Co. In addition, the demand for renewable energy will be nearly fifty times higher than that of fossil fuels energy, implying more diversified demands for various minerals. Finally, several policy recommendations are proposed to help improve the overall resource efficiency, such as strategic reserves, material substitutions, and circular economy.

***Keywords:* Carbon neutrality; Critical mineral; Mismatch gap; Power system; Circular economy**

1. **Introduction**

Over the past few decades, global efforts on combating climate change have been made, in which more than one hundred countries outlined their intentions to address climate change [1]. Based on the Paris Agreement, many countries have made their ambitious commitments to move toward low carbon development [2]. More than 120 countries have pledged to achieve carbon neutrality before 2050 [3]. In 2020, President Xi Jinping announced that China would increase its nationally determined contributions, prepare more effective policies and measures, strive to achieve a carbon emissions peak by 2030, and achieve carbon neutrality by 2060. As the entire power system is the major contributor to national carbon emissions (41.6% in 2019) [4], it is critical to promote energy transition to achieve these ambitious goals. In particular, replacing conventional fossil fuel-based energy with renewable energy is the key to optimizing the entire energy structure.

However, the development of renewable energy highly relies on critical mineral resources, which may induce concerns on potential minerals shortage and corresponding environmental emissions [5]. These critical minerals are essential for the construction and operation of national power systems. Moreover, the supply chains of these minerals are vulnerable because most of these minerals are highly concentrated in a few countries, and the geopolitical situations further exaggerate such risks [6].

Previous relevant studies mainly focus on mineral demand prediction and supply risk assessment for some specific technologies (such as photovoltaic solar technology [7], onshore and offshore wind power technology [8], electric vehicles battery storage technology [9]). Several studies uncover the potential resource constraints [10,11]. For example, Wang et al. [12] predicted eight types of critical mineral resources demand for solar power generation technology and found that there will be serious minerals shortage by 2050. Li et al. [8] found that the global demand for rare earth elements (REE) in the field of wind power generation will be imbalanced with their supplies in 2050. Also, these studies were conducted at the global dimension, rather than at national or regional levels [13-15]. In addition, such studies mainly aimed at low carbon development by employing scenarios analysis, without considering carbon neutrality targets [16,17]. Consequently, it is urgent to comprehensively assess critical mineral resources demand and supply for the power system by considering carbon neutrality targets.

Under such circumstances, this study aims to develop a comprehensive and systematic evaluation method to investigate the critical minerals constraints on Chinese power generation under different climate target scenarios. Ten climate target scenarios proposed by various research institutions were first collected to illustrate future demand structure and electricity mix trajectories. Energy sources, including solar power, wind power, hydropower, geothermal power, nuclear power, biomass, coal and natural gas were included in this study. Additionally, carbon capture and storage (CCS) projects for coal and natural gas power generation were considered as well, while wind and solar power projects were divided into onshore and offshore, photovoltaic (PV) and concentrated solar power (CSP) projects, respectively [18]. We then calculate the cumulative demands of these power systems for twenty-three types of critical minerals (International Energy Association (IEA) classification) in 2060 via dynamic material flow analysis (DMFA). We further evaluate different climate target scenarios, energy lifespans, and recycling potentials. The demand uncertainty is analyzed by considering the uncertainties in the power system and minerals demand structures of the different energy types. The results indicate that the current supply capacities of critical minerals in China cannot satisfy the demand under the different climate target scenarios, especially carbon neutrality-focused scenarios. Increasing strategic reserves, material substitution, and recycling secondary resources are efficient approaches to address these problems.

1. **Methods and data sources**

This study investigates critical mineral flows under different Chinese climate target scenarios until 2060 by using DMFA[19-20]. Ten climate target scenarios are considered, focusing on different energy transition pathways proposed by different research institutions. We divide these scenarios into four categories based on the characteristics of each scenario [21-26]. Twenty-three types of critical minerals are included in this study, covering relevant stocks and decommissioning aspects.

*2.1. Mineral flows in the Chinese power system*

DMFA is employed to estimate the installed capacity and decommissioning of the power system, focusing on three parameters: stocks, inflows, and outflows. Inflows and stocks reflect the accumulated installation of different power systems, while outflows represent power decommissioning. These outflows are accounted based on the historical inflows and power lifetime:

 (1)

 (2)

where *Outflow_p_* (*t_n_*) is the sum of the past inflows *t_i_* of decommissioned power system P from t_0_ to t_n-1_, *Survival(t)* denotes the complementary cumulative distribution function, which follows a normal distribution [8], *Outflow_total_* is the total decommissioning amount of the power system, which is summed up by all the outflows. According to the mass balance principle, the inflows equals to the changes in stocks plus all the outflows during this period.

The cumulative demand for critical minerals (*Inflow_m_(t_n_*)) in the power system is accounted by using Eq. 3：

 (3)

Where *Inflow_p_(t_n_)* represents the total material inflow from power system *p* in year *t_n_,* and the *coe_p,m_(t_n_)* is the material coefficient of element *m* in power system *p* in year *t_n_.*

The expansion ratio (Exp) is proposed in this study to compare the cumulative minerals demand in 2060 to the current minerals demand and can be calculated by using Eq. 4:

*Exp=Cumulative demand in 2060/Current demand in 2020* (4)

*2.2.* *Critical mineral reserves and production capacity*

Current production capacity of each critical mineral is u calculated by using Eq. 5:

** (5)

where *R_ij_* is the current production capacity of mineral *i* in year *j*, *F_i_* is the weight fraction for element *i*, *T_ik_* is the grade of element *i* in mine *k*, and *P_kj_* is the mineral production in mine *k* in year *j*. *L_k_* is the loss rate in mine *k* at the production stage. The estimated reserve amount of a given mine is calculated by using Eq.6:

 (6)

where *G_k_* is the critical mineral reserve in mine *k*.

*2.3. Sensitivity analysis*

The results of this study are subjected to uncertainties from various parameters. Thus, a sensitivity analysis is conducted to measure the uncertainties of these parameters on the final results. In this study, we explore the input uncertainty from two variables: power system structure and mineral demand structure. Since all of the parameters were obtained from research reports [21-26], we set the CVs in this study at 5% [27,28]. Indirect data were those data calculated from direct data, in which the CV values were calculated from Gauss’s law of error propagation. The results of sensitivity analysis for different mineral demands are illustrated in Figs. S1-S23, while the parameters settings for sensitivity analysis are listed in Table S7. These results indicate that they have marginal impacts on the key results of this study.

*2.4. Data sources*

Data used in this study were obtained from different sources. Ten climate target scenarios were retrieved from different research reports, including four scenarios from the International Energy Agency (IEA) (baseline development scenario (BDS), reference (Ref) scenario, 2 degrees scenario (2DS), and beyond 2 degrees scenario (B2DS)), three scenarios from Tsinghua University (strengthened ambition scenario (SAS), 2DS, and 1.5 degrees scenario (1.5DS)), two scenarios from the World Resources Institute (WRI) (state policy scenario (SPS) and SAS) and one scenario from the Global Energy Interconnection Development and Cooperation Organization (GEIDCO) (CNS). More details on these ten scenarios can be found in the supplementary material. The parameters considered in this study were also retrieved from these reports. Data on reserve amounts, production amounts, and coefficients were derived from the United States Geological Survey [29]. Since certain scenarios exhibited similar trends in electricity mix and generation, we combined these ten scenarios into four scenarios for further analysis: SPS, SAS, 2DS, and CNS. Twenty-three types of critical minerals, including Cd, Cu, Ga, In, Pb, Se, Si, Ag, Te, Sn, Zn, As, Cr, Mn, Mo, Ni, Mg, Ti, Nb, W, Zr, REE, and Co, were examined in this study [26].

1. **Results**

*3.1. Great differences in the minerals demand between ten climate scenarios*

Under the circumstance of energy transition in China, the potential power generation capacity will gradually increase from 2150 GW in 2020 to 3630–8010 GW in 2060 under ten different scenarios [21-26]. This means that the demands for critical minerals will increase from 10,576 kt in 2020 to 22,259–74,680 kt in 2060. Fig. 1 shows China’s potential power generation capacities and minerals demands in 2060, which are highly different under different scenarios. When the climate neutrality goals become more ambitious, the generation capacity of the power system will increase. Also, the electricity mix will shift toward renewable energy since low-carbon energy transition requires that conventional energy is substituted by renewable energy. GEIDCO defines CNS as the most ambitious scenario since this scenario targets to achieve zero emission in 2060. Under this scenario, coal will not be used for power generation, while most natural gas will be supplied by CCS projects [26,30].

The cumulative demand for these critical minerals until 2060 will experience similar trends under different scenarios. The cumulative demand for critical minerals will increase when more ambitious climate target is set up, while such demand for fossil fuels will decline accordingly. Although the total demand for such minerals is different under different scenarios, such demand structures are similar. Cu, Si, Zn, and Cr are key elements for the applications of most power generation technologies, accounting for more than 50% of the total critical minerals demand, with figures of 6526–31591 kt, 1489–11786 kt, 4797–20890 kt, and 1287–4679 kt by 2060, respectively. Such demands are 100–10000 times higher than those for other critical minerals, such as In, Ga, Co, and REE. Detailed minerals demands are listed in Tables S1-S5. By comparing the expansion ratio of the total demand to the current minerals demand (in 2020) driven by the current minerals production capacity, we found that current minerals production capacity should be increased by 2 to 7 times to meet the future demand. Moreover, it is unclear whether the total domestic reserves of these critical minerals can satisfy with the future demands. Since minerals production capacity depends on the reserves, insufficient reserves may lead to uncertainty in the corresponding supply chains.

Since these scenarios present similar conditions in terms of the installed power generation capacity and cumulative minerals demand, we combine them into four scenarios, namely, SPS, SAS, 2DS, and CNS. SPS scenario means that current policy framework will be maintained, including the achieved targets under the 14^th^ Five-Year Plan. SAS scenario represents that climate policy will be more ambitious. 2DS scenario means that temperature growth will be restricted within 2 degrees in China until 2060. CNS scenario is the most ambitious scenario in this study, indicating that China will achieve carbon neutrality by 2060,

*3.2. High and complicated minerals demand from renewable energy development*

The cumulative demands for critical minerals under four scenarios in 2060 are shown in Fig. 2, covering solar power, coal, natural gas, wind power, hydro-power and nuclear power technologies. It is clear that the critical minerals demands from renewable energy are much higher than those from conventional energy. The total critical minerals demand from renewable energy ranges from 2,898 to 38,743 kt by 2060, nearly fifty times the demand from conventional energy (ranging from 403 to 800 kt under different scenarios). Also, minerals demand structures for wind power, hydro-power and nuclear power are much more diversified than those for conventional energy, such as coal and natural gas. For example, minerals demand for wind power involves in many elements, including Cu, Zn, Cr, Mn, Mo, Ni and several rare earth elements. However, minerals demand for solar power is relatively simple, in which Cu and Nb dominate and account for more than 80% of the total demand. In addition, Cu is the most critical resource for conventional power generation technologies and accounts for more than 50% of the total demand, followed by Zn and REE. In general, more than 50% of such critical minerals for the application of renewable energy technologies are other minerals, such as As, Mn, and Mo.

The cumulative demands for critical minerals from different power generation technologies will experience different trends under these four scenarios. Such minerals demand from the application of renewable energy power generation technologies will increase when the climate goals become more ambitious (wind power: from 9763 to 38743 kt; solar power: from 4198 to 26592 kt; hydropower: from 3780 to 5320 kt). In contrast, such minerals demand from the application of fossil fuels-based power system will experience opposite trends, especially under the CNS scenario. For instance, the critical minerals demand from coal-fired power generation is zero.

*3.3. Significant mismatch between the cumulative demands, production capacities, and reserves*

We compared minerals reserves, cumulative minerals production amounts, and cumulative minerals demands based on the current production capacities under the above four scenarios. The results indicate that current production capacities and reserves cannot meet the future demands for most minerals. The gap between minerals demand and supply will continuously expand in the future. These critical minerals can be divided into three categories.

The first category is the superior resource category, where sufficient reserves exist in China. The cumulative minerals production capacity can support power generation demand from now until 2060, including Pb, Cd, In, Se, Si, Zn, As, Ti, W, Zr, and REE. For example, compared to 2020, the expansion ratio of Pb ranges from 1.65 to 2.23, and the cumulative Pb demand ranges from 267 to 484 kt under different scenarios. Based on the current production capacity, the cumulative Pb production amount will reach approximately 6,000 kt in 2060, while Pb reserve is 6,052 kt in China. Therefore, China does not need to worry about such minerals, including Pb, Si, Zn, and Ti [31].

The second category refers to those minerals with abundant reserves but without sufficient production capacities, such as Sn, Mg, Nb, and Mo. Their reserves are rich in China, but their current production capacity cannot meet the future demands under certain ambitious scenarios. The main reason is that the current production capacity is controlled by the current demand level [32]. With the increasing demands, such production capacity should simultaneously be increased to satisfy future demands. For example, China is a leading supplier of Mg and has established a holistic supply chain from mining to the final products, but the current production quota restricts its production capacity [33]. Thus, such production capacity should be enhanced to meet future demands.

The third category includes Cu, Ga, Mn, Te, Cr, Ag, Co, and Ni. Unfortunately, China does not have adequate reserves for such minerals, and the corresponding production capacity is low, leading to that China has to depend on importing them from other countries. According to the scenarios analysis results, Cu accounts for approximately 50% of the total mineral demand, ranging from 6,526 to 31,596 kt. In contrast, Cu cumulative production and reserve will be only 486 and 26,800 kt in 2060, respectively. More than 80% of such minerals have to be imported to meet the domestic demands [34]. The corresponding expansion ratios range from 0.95 to 3.44 and 2.12 to 7.09, respectively. Such results indicate that it is critical for the Chinese government to prepare appropriate critical minerals management strategy to facilitate energy transition so that ambitious carbon neutrality target can be achieved.

1. **Discussion**

*4.1. Challenges in the critical minerals supply chain*

Since China does not have adequate reserves of certain critical minerals, such as Cu, Ni, Cr, and Co, China must purchase them from other countries to support its energy transition. Unlike fossil fuels such as coal and natural gas, minerals supply for renewable energy development is more concentrated. Taking Ni, Co, and REE as examples, the top three production countries have dominated more than three quarters of the global exports. In particular, one single country may be responsible for approximately half of the global production [21]. For example, China supplied more than 60% of rare earth elements globally in 2019 although its reserves only account for 38% of the global reserves [35]. The manufacturing of such minerals is even more concentrated, leading to higher supply risks, such as physical disruption, trade restrictions, or other issues in major producing countries. For example, the recent trade disputes between China and the United States may induce future trade uncertainties, indicating potential minerals supply constraints may hinder energy transition in the power system [36]. Consequently, it would be necessary to establish strategic minerals reserve bases across the whole country to ensure that such minerals supply will be stable so that energy transition can be achieved in due time. Moreover, international cooperation is also crucial since no single country can meet such increasing minerals demands by itself.

*4.2. Efficiency improvement via circular economy*

Since many countries have released their ambitious climate targets have been proposed over the past few years, now these critical minerals deserve more attentions. Especially, several countries have formulated relevant trade protection policies, leading to unstable supply chains [37-39]. Also, since mining projects are both capital and emission intensive, many countries with such reserves tend to protect their mineral resources rather than mine them [40]. In order to solve such a problem, it is critical to seek alternative minerals, such as secondary resource from urban mining through the implementation of circular economy. [41]. Table S6 lists the typical lifespans of different power generation technologies. It is clear that most technology options have a lifespan between 20 and 40 years, except nuclear power. Thus, newly installed projects will gradually retire from 2050 to 2060. It is estimated that the total secondary resource of Cu, Pb, Si, Zn, Cr, and Mn from decommissioning will reach 4,373, 216, 775, 2,393, 1,360, and 434 kt, respectively [26]. If the recycling rates for such elements exceed 80%, then approximately 30%-50% of the critical mineral demands under SPS, 16%-30% under SAS, 8%-16% under 2DS, and 4%-12% under CNS can be achieved. However, the current recycling rates of these critical minerals only range from 30% to 40% [42,43]. Hence, it is essential to improve the recycling rates through circular economy. However, several challenges exist since recycling activities are always associated with high costs and technological and institutional barriers [44]. Also, the environmental impacts of such recycling activities cannot be ignored, such as additional energy consumption and corresponding greenhouse gas emissions and other environmental emissions. Therefore, governments at different levels should prepare more feasible policies to facilitate the implementation of circular economy, such as financial subsidies, capacity building efforts, research and development support, and information sharing.

*4.3. Material substitutions with technological support*

The expansion ratios of different critical minerals in this study range from 1.13 to 6.94 under SPS, from 1.42 to 8.67 under SAS, from 1.55 to 9.45 under 2DS, and from 1.18 to 15.2 under CNS, indicating that it would be difficult to meet with such demands if only relying on the current production capacities and reserves. Since it is not easy to fully engage in minerals recycling, it is therefore critical to improve material efficiency through technological improvement. For instance, it is possible to seek substitute materials to replace conventional minerals. Renewable energy demand for critical minerals is more diversified than conventional energy demand. In this regard, fifteen and eight critical minerals are essential for solar and wind power generation, respectively, while coal-based and natural gas-based power generation only rely on five and three critical minerals, respectively [21]. This means that it is more difficult to identify appropriate substitute materials for the application of renewable energy technologies. Fortunately, manufacturers have already made their efforts to replace different materials and adjust corresponding minerals structures for energy power systems, resulting in lower demands for such critical minerals [45-46]. However, more assessments are needed to ensure that such substitutions can keep the stable operation of energy systems.

*4.4. Mineral demands for power transmission and storage*

A complete power system should include power transmission and storage projects rather than only power generation [47]. However, studies on the mineral demands for power transmission and storage are still lacking. Usually, power generation places are far away from power consumption places, thus many power transmission projects have been finished to deliver electricity. In 2017, transmission lines with voltage classes over 220 kV reached a length of 6.87 × 10^5^ km in China, approximately twice the length of those in Europe [48]. It is expected that such lines will be further extended over the next 30 years. However, the construction of power transmission infrastructure relies on critical resources, such as steel/iron, aluminum, and many other critical minerals [49].

Moreover, critical minerals demands for power storage deserve more attention. Power storage is essential to mitigate fluctuations and ensure dispatchable renewable energy sources [50-51]. Current studies mainly focus on construction scales, but many disputes exist since these studies used different parameters or scenarios. For example, the World Bank predicted that the cumulative minerals demand for power storage would reach 260 million tons in 2060 globally. Graphite, which is not covered in this study, accounts for over 50% of the global cumulative demand. Other critical minerals, such as Li, Pb, Ni, and Mn, are also major critical minerals for such a purpose [26]. Therefore, it is urgent to initiate more studies to evaluate the criticality of such minerals so that power storage can be achieved smoothly.

*4.5. Policy implications on critical minerals management*

Based on the above results, several policy recommendations are proposed. First, considering the potential supply-demand imbalance of critical minerals under different scenarios, it is vital for the Chinese government to consider the strategic reserve of such scarce minerals. Currently, specific storage policies on critical minerals are still lacking in China. It is necessary to establish minerals reserve bases. Also, it is expected that China will diversify its minerals supply by considering both domestic and international markets, which will help maintain a stable global supply chain. In this regard, international cooperation is crucial so that unnecessary stockpiles can be avoided and minerals prices can be kept relatively stable. Second, it is urgent to improve material efficiency and seek appropriate substitution materials. Both governments and companies should work together to allocate adequate research funds to support the development of advanced technologies. Meanwhile, the whole industrial process should be optimized to improve material efficiency and reach an economy of scale from the life cycle view. Finally, since many on-going power projects will retire in the future, indicating that more secondary resources will be available from such End-of-Life (EoL) products. Thus, the Chinese government should actively encourage recycling activities through the promotion of circular economy so that more critical minerals can be recovered from such EoL products. Necessary policies should be prepared to facilitate such recycling efforts, such as the establishment of regional EoL products collection centers, financial subsidies and national information system on critical minerals.

**5 Conclusions**

This study predicts the cumulative minerals demand for the Chinese power system until 2060 by using DMFA. Minerals production amounts, reserves, and demands for the application of different energy technologies are compared under ten different scenarios. The results under different scenarios are significantly different. The total minerals demand will increase when the climate target becomes more ambitious. Especially, minerals demands for renewable energy are much higher and more diversified than those for conventional energy. Also, a significant mismatch may occur between the future minerals demand and supply. Current minerals production capacity could not meet with future minerals demand. Based upon such results, several policy suggestions are raised, including the implementation of circular economy, material substitutions, strategic reserves, and international cooperation.

However, several research limitations exist. First, we only investigated the minerals demands for power generation, but not for power transmission and storage. Second, it would be ideal to consider the use of such minerals in other fields so that a more holistic perspective can be obtained. Such a holistic consideration can help accurately assess minerals supply and demand so that more appropriate policy insights can be obtained. Finally, more regional studies should be initiated to prepare more region-specific policies by considering the local realities. These efforts could be future research directions.

**Declaration of Competing Interest**

The authors declare that they have no conflicts of interest in this work.

**Acknowledgments**

This work was supported by the National Key R&D Program of China (2019YFC1908501), the National Natural Science Foundation of China (Grants No. 72088101, 71904125, 71810107001, and 71690241)

**References**

[1] J. Rogelj, W. Hare, J. Lowe, et al., Emission pathways consistent with a 2 °C global temperature limit. Nat. Clim. Chang 1 (2011) 413-418.

[2] J. Rogelj, E. M. Den, N. Höhne, et al., Paris Agreement climate proposals need a boost to keep warming well below 2 °C. Nature 534 (2016) 631-639.

[3] S. K. Gallagher, F. Zhang, R. Orvis, et al., Assessing the policy gaps for achieving China's climate targets in the Paris Agreement. Nat. Commun. 10 (2019) 1256.

[4] G. He, J. Lin, F. Sifuentes, et al., Rapid cost decrease of renewables and storage accelerates the decarbonization of China's power system. Nat. Commun. 11 (2020) 2486.

[5] K. Tokimatsu, M. Höök, B. McLellan, et al. Energy modeling approach to the global energy-mineral nexus: Exploring metal requirements and the well-below 2 °C target with 100 percent renewable energy. Appl. Energy 225 (2018) 1158-1175.

[6] D. Bogdanov, J. Farfan, K. Sadovskaia, et al., Radical transformation pathway towards sustainable electricity via evolutionary steps. Nat. Commun. 10 (2019) 1077.

[7] C. Helbig, A. M. Bradshaw, C. Kolotzek, et al., Supply risks associated with CdTe and CIGS thin-film photovoltaics. Appl. Energy 178 (2016) 422-433.

[8] J. Li, K. Peng, P. Wang, et al., Critical rare-earth elements mismatch global wind-power ambitions. One Earth 3 (2020) 116-125.

[9] V. V. Klimenko, S. V. Ratner, A. G. Tereshin. Constraints imposed by key-material resources on renewable energy development. Renew. Sust. Energ. Rev. 144 (2021) 111011.

[10] Y. Zhu, J. Xie, A. Pei, et al., Fast lithium growth and short circuit induced by localized-temperature hotspots in lithium batteries. Nat. Commun. 10 (2019) 2067.

[11] P. Greim, A. A. Solomon, C. Breyer, Assessment of lithium criticality in the global energy transition and addressing policy gaps in transportation. Nat. Commun. 11 (2020) 1-11.

[12] P. Wang, L. Che, J. Ge, et al. Incorporating critical material cycles into metal-energy nexus of China’s 2050 renewable transition. Appl. Energy 253 (2019) 113612.

[13] G. Kalt, P. Thunshirn, D. Wiedenhofer, et al. Material stocks in global electricity infrastructures - An empirical analysis of the power sector's stock-flow-service nexus. Resour. Conserv. Recycl. 173 (2021) 105723.

[14] F. Lozano, M. Ortiz, R. Lozano, et al. New perspectives for green and sustainable chemistry and engineering: Approaches from sustainable resource and energy use, management, and transformation. J. Clean. Prod. 172 (2018) 227-232.

[15] P. Capros, G. Zazias, S. Evangelopoulou, et al., Energy-system modelling of the EU strategy towards climate-neutrality. Energy Policy 134 (2019) 110960.

[16] F. Li, Z. Ye, X. Xiao, et al. Material stocks and flows of power infrastructure development in China. Resour. Conserv. Recycl. 160 (2020) 104906.

[17] P. Viebahn, O. Soukup, S. SAmadi, et al. Assessing the need for critical minerals to shift the German energy system towards a high proportion of renewables. Renew. Sust. Energ. Rev. 49 (2015) 655-671.

[18] N. Kittner, F. Lill, D. M. Kammen, Energy reserve deployment and innovation for the clean energy transition. Nat. Energy 2 (2017) 17125.

[19] F. K. Marina, The intellectual history of materials flow analysis, part I, 1860-1970, J. Ind. Ecol. 2 (1998) 61-78.

[20] F. K. Marina, The intellectual history of materials flow analysis, part II, 1970-1998, J. Ind. Ecol. 2 (1998) 107-136.

[21] International Energy Agency, The role of critical minerals in clean energy transitions. https://www.iea.org/reports/the-role-of-critical-minerals-in-clean-energy-transitions/, 2021 (accessed 1 May 2021).

[22] World Resources Institute, Accelerating the net-zero transition: strategic action for China’s 14^th^-five-year plan. https://www.wri.org/research/accelerating-net-zero-transition-strategic-action-chinas-14th-five-year-plan/, 2020 (accessed 3 December 2020).

[23] Institute of Climate Change and Sustainable Development of Tsinghua University. China's long-term low-carbon development strategy and transition pathway. China Population Resources and Environment 30 (2020) 1-25.

[24] Global Energy Interconnection Development and Cooperation Organization, Report on China's carbon neutrality by 2060. https://www.geidco.org.cn/html/qqnyhlw/zt20210120_1/index.html/, 2021 (accessed 18 March 2021).

[25] N. Zhou, H. Lu, N. Khanna, et al., China Energy Outlook: Understanding China’s Energy and Emissions Trends, Berkeley, Lawrence Berkeley National Laboratory, 2020.

[26] The World Bank Group, Minerals for Climate Action: The mineral intensity of the clean energy transition. https://pubdocs.worldbank.org/en/961711588875536384/Minerals-for-Climate-Action-The-Mineral-Intensity-of-the-Clean-Energy-Transition.pdf, 2020 (accessed 11 May 2020).

[27] Q. Liu, Z. Cao, X. Liu, et al. Product and metal stocks accumulation of China’s megacities: patterns, drivers, and implications. Environ. Sci. Technol. 53 (2019) 4128-4139.

[28] S. Hand, X. Shang, J. Guest, et al. Global sensitivity analysis to characterize operational limits and prioritize performance goals of capacitive deionization technologies. Environ. Sci. Technol. 53 (2019) 3748-3756.

[29] U.S. Geological Survey. Data sheet: Mineral commodity summaries 2020. 2020, https://pubs.usgs.gov/periodicals/mcs2020/.

[30] J. A. Mohammad, S. C. Cao, J. Jung, Geological CO_2_ sequestration in saline aquifers: Implication on potential solutions of China's power sector. Resour. Conserv. Recycl. 121 (2017) 137-155.

[31] J. L. Reyna, M. V. Chester, Energy efficiency to reduce residential electricity and natural gas use under climate change. Nat. Commun. 8 (2017) 14916.

[32] D. D. Imholte, R.T. Nguyen, A. Vedantam, et al., An assessment of U.S. rare earth availability for supporting U.S. wind energy growth targets. Energy Policy 113 (2018) 294-305.

[33] S. Ramakrishnan, P. Koltun, Global warming impact of the magnesium produced in China using the Pidgeon process. Resour. Conserv. Recycl. 42 (2004) 49-64.

[34] J. Dunn, M. Slattery, A. Kendall, Circularity of Lithium-Ion battery materials in electric vehicles. Environ. Sci. Technol. 55 (2021) 5189-5198.

[35] Q. Wang, P. Wang, Y. Qiu, et al., Byproduct surplus: lighting the depreciative europium in China's rare earth boom. Environ. Sci. Technol. 54 (2020) 14686-14693.

[36] Y. Guo, A. Hawkes, The impact of demand uncertainties and China-US natural gas tariff on global gas trade. Energy 175 (2019) 205-217.

[37] R. D. Leal-Ayala, J. M. Allwood, E. Petavratzi, et al., Mapping the global flow of Tungsten to identify key material efficiency and supply security opportunities. Resour. Conserv. Recycl. 103 (2015) 19-28.

[38] N. A. Mancheri, World trade in rare earths, Chinese export restrictions, and implications. Resources Policy 46 (2015) 262-271.

[39] G. Luderer, M. Pehl, A. Arvesen, et al., Environmental co-benefits and adverse side-effects of alternative power sector decarbonization strategies. Nat. Commun. 10 (2019) 5229.

[40] S. W. Harpole, L. L. Sullivan, E. M. Lind, et al., Addition of multiple limiting resources reduces grassland diversity. Nature 537 (2016) 93-96.

[41] X. Zeng, S. H. Ali, J. Tian, et al., Mapping anthropogenic mineral generation in China and its implications for a circular economy. Nat. Commun. 11 (2020) 1544.

[42] T. E. Graedel, J. Allwood, J. Birat, et al. What do we know about metal recycling rates? J. Ind. Ecol. 15 (2011) 355−366.

[43] J. Wang, Y. Ju, M. Wang, et al. Scenario analysis of the recycled copper supply in China considering the recycling efficiency rate and waste import regulations. Resour. Conserv. Recycl. 146 (2019) 580-589.

[44] D. Raabe, C. C. Tasan, E. A. Olivetti, Strategies for improving the sustainability of structural metals. Nature 575 (2019) 64-74.

[45] R. Lin, M. Liu, Q. Wu, et al., Anomalous metal segregation in Lithium-rich material provides design rules for stable cathode in Lithium-ion battery. Nat. Commun. 10 (2019) 1650.

[46] H. Hao, Y. Geng, J. E. Tate, et al., Impact of transport electrification on critical metal sustainability with a focus on the heavy-duty segment. Nat. Commun. 10 (2019) 5398.

[47] S. Agnew, P. Dargusch, Effect of residential solar and storage on centralized electricity supply systems. Nat. Clim. Chang 5 (2015) 315-318.

[48] W. Wei, J. Li, B. Chen, et al., Embodied greenhouse gas emissions from building China's large-scale power transmission infrastructure. Nat. Sustain. 4 (2021) 739-747.

[49] K. Cheng, Y. Wang, H. Tian, et al., Atmospheric emission characteristics and control policies of five precedent-controlled toxic heavy metals from anthropogenic sources in China. Environ. Sci. Technol. 49 (2015) 1206-1214.

[50] F. P. Wyper, S. K. Antiochos, D. C. Richard, A universal model for solar eruptions. Nature 544 (2017) 452-455.

[51] S. Lu, R. Wang, Q. Lu, et al., Magnetotail reconnection onset caused by electron kinetics with a strong external driver. Nat. Commun. 11 (2020) 5049.
